# Supplementary material for: Analysis of asymptomatic Drosophila models for ALS and SMA reveals convergent impact on functional protein complexes linked to neuro-muscular degeneration
Source: BMC Genomics. 2023 Sep 27;24:576. doi: 10.1186/s12864-023-09562-4 (PMC10523761; doi:10.1186/s12864-023-09562-4)

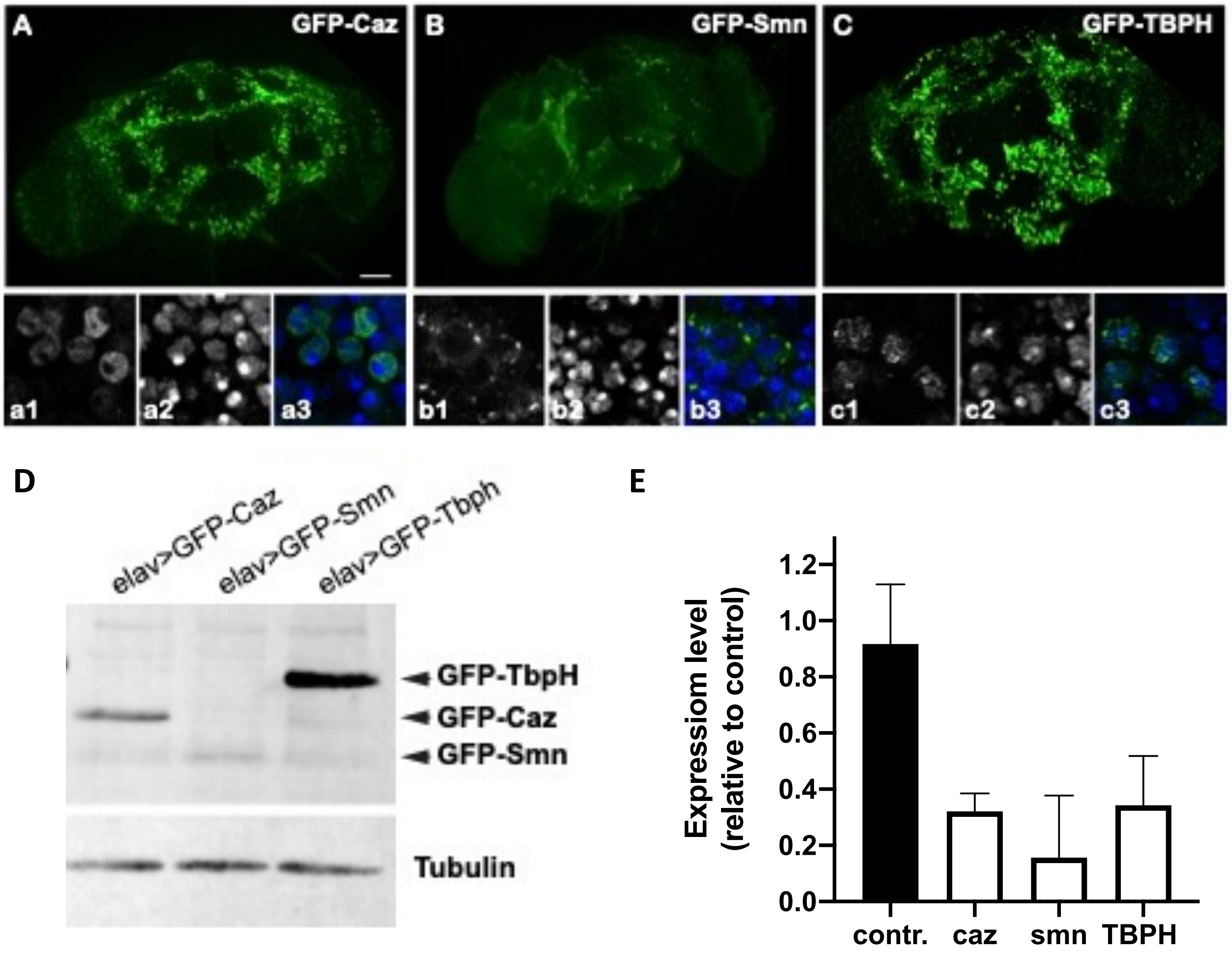

Supplementary Figure S1. Characterization of the UAS-GFP-Caz, UAS-GFP-Smn and UAS-GFP-TBPH fly lines. (A-C) Adult brains dissected from *elav*>GFP-caz (A), *elav*>GFP-Smn (B) and *elav*>GFP-TBPH (C) flies 5-7 days after expression. The GFP signal is shown in green. Insets in a1-a3, b1-b3 and c1-c3 show the sub-cellular distribution of the GFP-tagged proteins. GFP signals are shown in white (left) or green (overlay, right). DAPI signals are shown in white (middle) or blue (overlay, right). Scale bar: 50 µm. Complete genotypes: *elav*-Gal4/Y; tub-Gal80ts/UAS-GFP-*caz* (A), *elav*-Gal4/Y; tub-Gal80ts/UAS-GFP-*Smn* (B) and *elav*-Gal4/Y; tub-Gal80ts/UAS-GFP-*TBPH* (C). (D) Western blot performed on lysates from adult flies with pan neural (*elav*-Gal4) expression of GFP-Caz (left), GFP-Smn (middle) and GFP-TBPH (right) brains. Anti-GFP antibodies were used to detect GFP fusions. Tubulin was used as a loading control. (E) qRT-PCR quantification of caz, Smn, and TBPH in VDRC strain #13673 expressing dsRNA targeting *always early* (contr.) and flies containing the shRNA transgene for each target gene and grown in the presence of hormone for 10 days. ddCt values to the endogenous control gene Rp49 were normalized to the corresponding ddCts from samples of flies grown in the absence of hormone. The black bar for the control samples represents the average normalized expression of the three target genes in the VDRC strain.

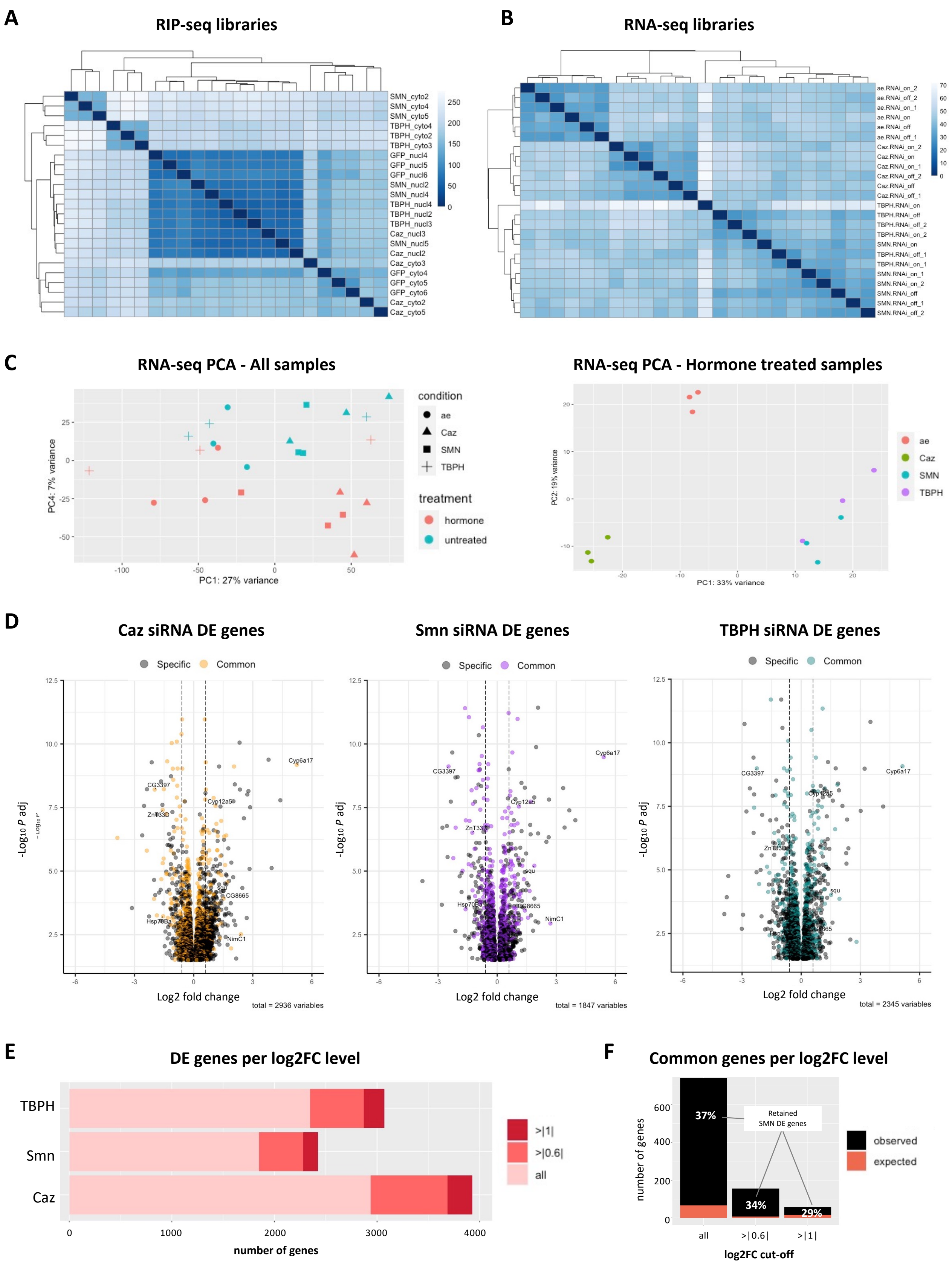

Supplementary Figure S2. Overview of sequencing datasets. Sample-to-sample distance heatmap for (A) the RIP-seq and (B) the RNA-Seq datasets, revealing overall similarities and dissimilarities between dataset samples based on Euclidean distance. (C) Principal component analysis for RNA-Seq datasets. Left: full dataset, samples colored by treatment, symbols indicate fly line (condition). Right: analysis of hormone treated samples, colored by fly line. (cont. in next page)

Supplementary Figure S2. (cont) (D) Volcano plots of DE genes identified for each knockdown (with adj. p value < 0.05). Genes displaying common changes across the three fly lines are highlighted in color. (E) Total number of DE genes identified in each sample type at increasing levels of | log2 FC |. (F) Number of common genes between the three knockdowns identified at increasing | log2 FC | cut-offs. The expected versus observed number of common genes in the overlaps is displayed in red and black, respectively. The percentage represented by the common genes in the smallest dataset of the overlap (Smn DE) is shown over the plot bars. Increasing fold change cut-offs leads to a progressively bigger reduction of "captured" genes without benefits to the functional enrichment analysis.

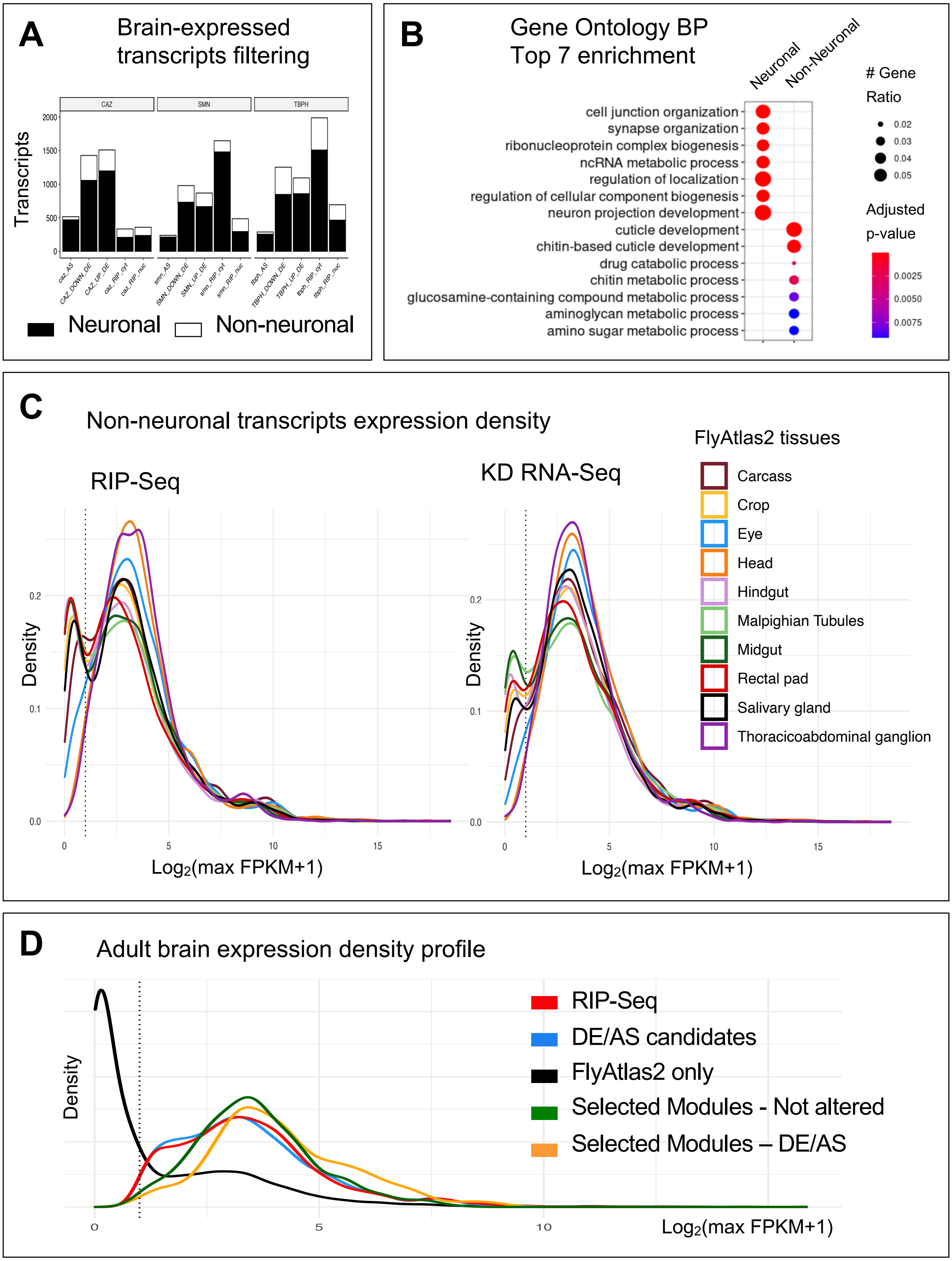

Supplementary Figure S3: Coverage of RIP-Seq and RNA-Seq experiments in FlyAtlas tissue-specific expression profiles. (A) Normalized RNA-Seq data of adult fly brain tissue was retrieved from the FlyAtlas2 database (see methods). The total 9020 transcripts were filtered using an expression threshold of > 1 FPKM. From the total 7369 transcripts identified in the RIP-Seq and knockdown experiments, 5511 were also detected in this dataset, and will be referred to as "neuronal" transcripts hereafter. Bar graph shows the number of transcripts identified in each experiment. (cont. in next page)

Supplementary Figure S3: Coverage of RIP-Seq and RNA-Seq experiments in FlyAtlas tissue-specific expression profiles. (cont.) (B) Figure summarizes the top 7 functions enriched in the sets of neuronal and non-neuronal transcripts identified in RIP-Seq and knockdown experiments. clusterProfiler R package was used to compare the functional enrichment of the 5511 “neuronal” and 1858 “non-neuronal” transcripts using Gene Ontology Biological Process, hyper-geometric test, adjusted p-value 0.05. From 824 enriched terms in neuronal transcripts, 92 include at the description the following key words: "synap", "axon", "neuro", "dendrite", "nervous", "button", "glial" or "cortex". Non-neuronal transcripts were enriched in 19 terms, none of them related to neuronal processes. (C) Figure shows density plots of log_2-transformed FPKM values for transcripts identified in the RIP-Seq and RNA-Seq experiments and classified as “non-neuronal”. 67.4% of the 1858 transcripts were detected in 10 additional tissues available in the FlyAtlas2 and displayed highest expression densities on *head*, *thoracicoabdominal ganglion* and *eye* tissues, explaining their possible origin in the datasets. (D) Density plot of log_2-transformed FPKM values of “neuronal” transcripts from the FlyAtlas2, RIP-Seq, DE/AS, and selected functional modules subsets, revealing an enrichment of our datasets in transcripts with medium to high expression levels in neurons, particularly for the transcripts with altered expression retained in the selected modules.

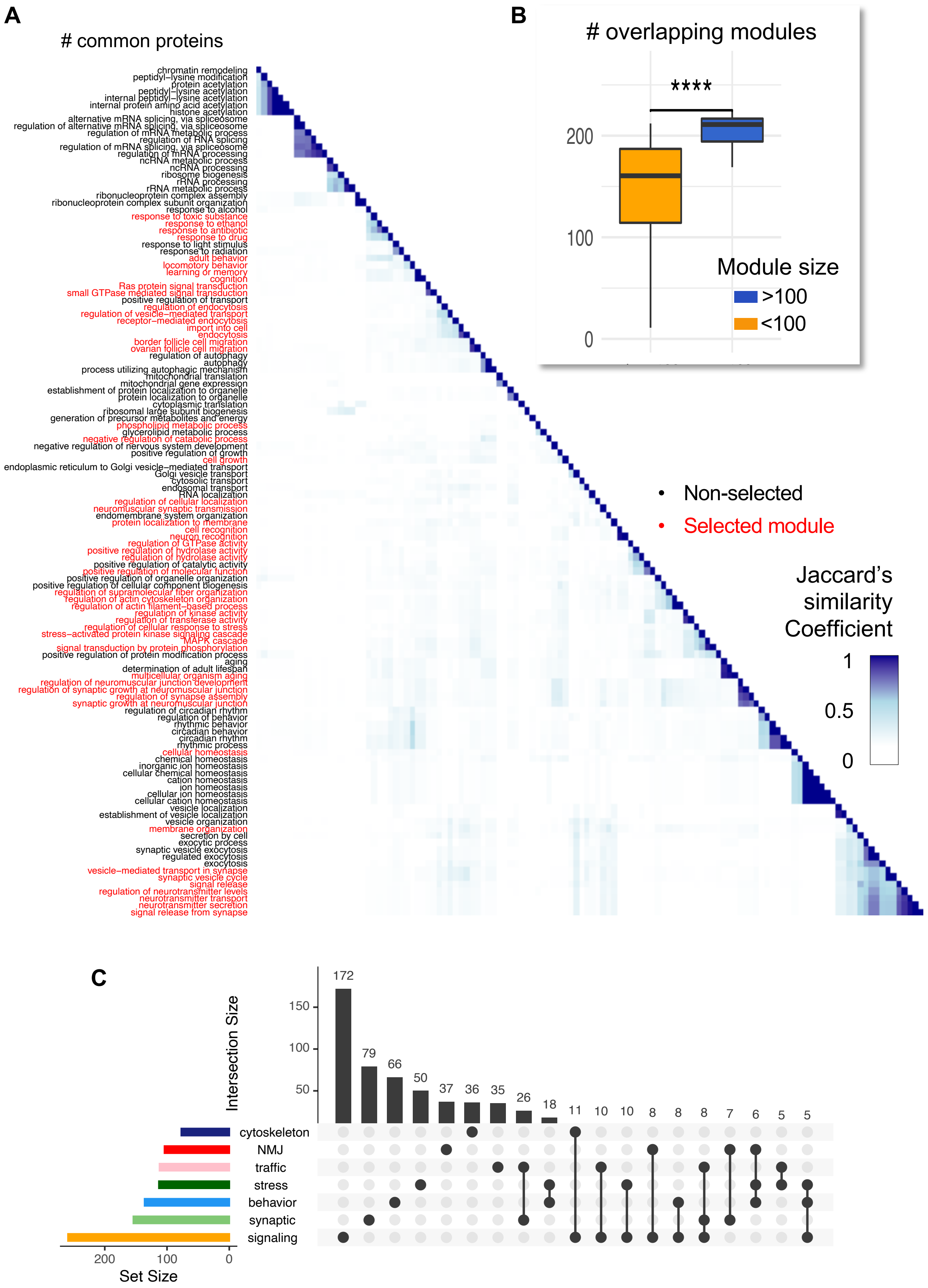

Supplementary Figure S4: Evaluation of protein redundancy across functional modules. (A) Complete-linkage hierarchical clustering using Jaccard's similarity coefficient for the 122 modules with a size between 10 to 100 proteins. The 52 modules passing the overall impact cut-off of >20% of transcripts altered in at least one knockdown are labeled in red. (cont. in next page)

Supplementary Figure S4: Evaluation of protein redundancy across functional modules. (cont.) (B) Box plots describing the number of modules sharing at least one protein when comparing modules including less or more than 100 proteins Wilcoxon test, p value 2.2×10^{-16}. (C) Bar plot indicating the number of proteins found in common between different super-modules. Colored horizontal bars indicate total number of proteins in each super-module. Black vertical bars indicate the overlap between super-modules. Only overlap sets including at least 5 proteins are shown.

Supplementary Methods – Primer sequences

**Primer name**	**Sequence (5'-3')**
Smn_fwd_cloning	CACCATGTCCGACGAGACGAACG
Smn_rev_cloning	GATGGAATTACTTCTTGGGTGTC
Caz fwd_cloning	CACCATGGAACGTGGCGGTTATGGTG
Caz_rev_cloning	TTAATATGGTCTCGAGCGCATGC
NotI_TBPH-fwd	AAAAGCGGCCGCCATGGATTTCGTTCAAG
XhoI_TBPH_rev	AAAACTCGAGTTAAAGAAAGTTTGACTTCTCCGC
RT-PCR caz_rev	TCCGCGATCGAAGCGACCTCC
RT-PCR caz_for	TCCTACGGAAATGGAGGCGCC
RT-PCR TBPH_for	GGAAGGGGCCGAATAACCCGAAC
RT-PCR TBPH_rev	CACACATCATTGGGTGACAGGCACC
RT-PCR Smn1_ for	AAGAAGAATGCCACAACTCCC
RT-PCR Smn1_ rev	CAATGGACGTAATAGTAGCTGGG
RT-PCR rp49_for	TCGGATCGATATGCTAAGCTGTCGCAC
RT-PCR rp49_rev	AGGCGACCGTTGGGGTTGGTGAG
RT-PCR act5C_for	CACACCGTGCCCATCTACGAG
RT-PCR act5C_rev	CTTCTGCATACGGTCGGCGATGC

Source data for Figure 1 – Garcia-Vaquero et al

File names:

Lamin_Tubulin.tif

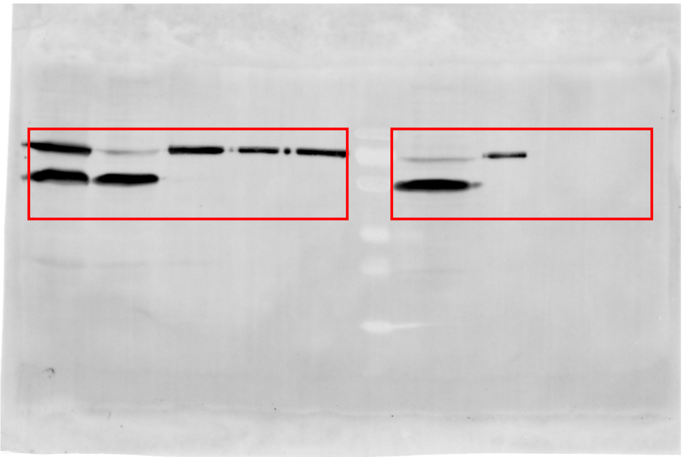

CAZ.tif

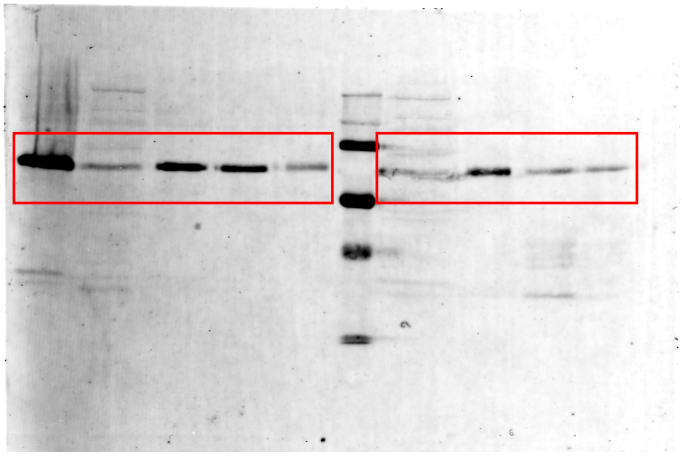

SMN.tif

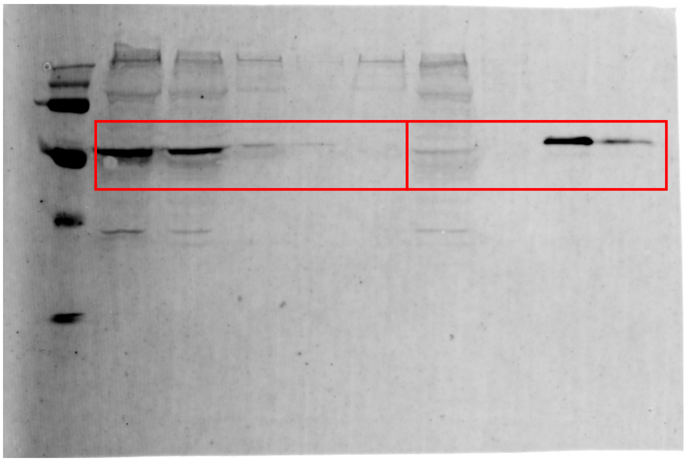

TBPH.tif

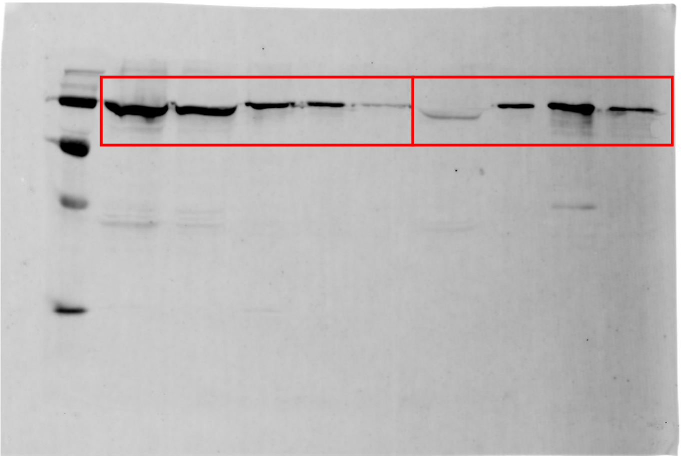

Source data for Supplementary Figure S1 – Garcia-Vaquero et al

File names:

GFP.tif

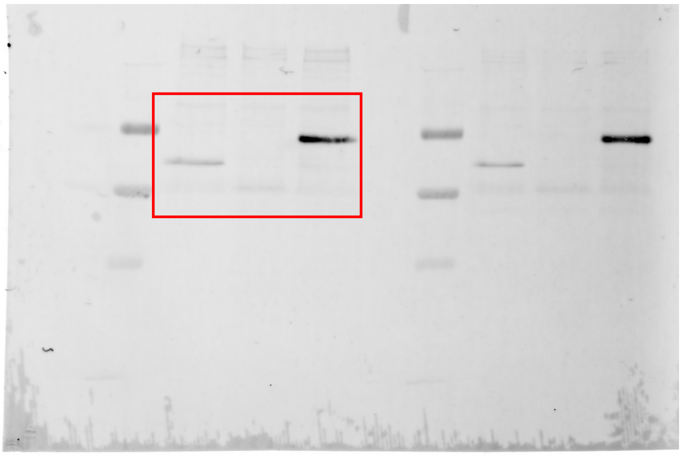

TUB.tif

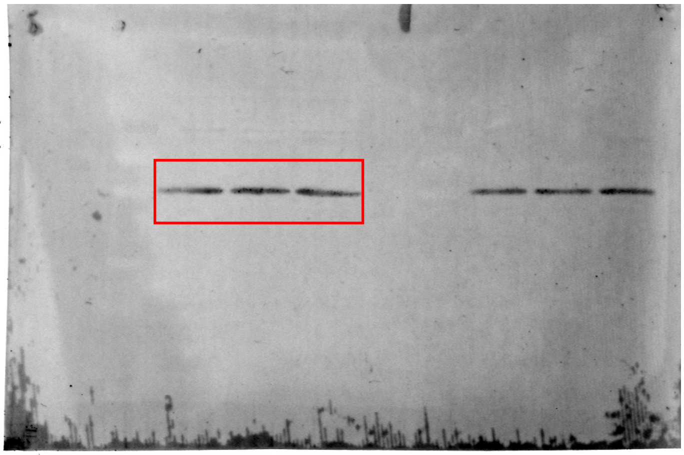

Supplement: Supplementary file 9 — Additional file 9. Supplementary Information. [file 12864_2023_9562_MOESM9_ESM.pdf]
